# The Reporting Frequency of Ketoacidosis Events with Dapagliflozin from the European Spontaneous Reporting System: The DAPA-KETO Study

**DOI:** 10.3390/ph15030286

**Published:** 2022-02-25

**Authors:** Gabriella di Mauro, Annamaria Mascolo, Mario Gaio, Concetta Rafaniello, Antonella De Angelis, Liberato Berrino, Giuseppe Paolisso, Francesco Rossi, Annalisa Capuano

**Affiliations:** 1 Campania Regional Centre for Pharmacovigilance and Pharmacoepidemiology, 80138 Naples, Italy; gabriella.dimauro@unicampania.it (G.d.M.); mario.gaio@unicampania.it (M.G.); concetta.rafaniello@unicampania.it (C.R.); francesco.rossi@unicampania.it (F.R.); annalisa.capuano@unicampania.it (A.C.); 2 Section of Pharmacology “L. Donatelli”, Department of Experimental Medicine, University of Campania “Luigi Vanvitelli”, 80138 Naples, Italy; antonella.deangelis@unicampania.it (A.D.A.); liberato.berrino@unicampania.it (L.B.); 3 Department of Advanced Medical and Surgical Sciences, University of Campania “Luigi Vanvitelli”, 80138 Naples, Italy; giuseppe.paolisso@unicampania.it; 4 Mediterrannea Cardiocentro, 80138 Naples, Italy

**Keywords:** dapagliflozin, ketoacidosis, diabetes mellitus, safety, adverse drug reaction

## Abstract

Dapagliflozin was associated with an increased risk of diabetic ketoacidosis that has led to the European withdrawal of the authorization for the type 1 diabetes. However, it is still used for the treatment of type 2 diabetes. Therefore, we aim to evaluate the occurrence of dapagliflozin-induced ketoacidosis events by using the European spontaneous reporting system. The reporting odds ratios (ROR) were computed to assess the reporting frequency of ketoacidosis events for dapagliflozin compared to Dipeptidyl peptidase-4 (DPP-4) inhibitors, insulins, or all other Sodium-glucose cotransporter-2 (SGLT-2) inhibitors. A total of 2406 cases with dapagliflozin reported at least one event of ketoacidosis. The three most reported events were: diabetic ketoacidosis (1412; 55.39%), ketoacidosis (476; 18.67%), and euglycaemic diabetic ketoacidosis (296; 11.61%). Dapagliflozin was associated with the higher reporting frequency of ketoacidosis events compared to DPP-4 inhibitors (ROR 12.07, 95%CI 11.67–13.81) or insulins (ROR 7.59, 95%CI 7.13–7.89). A lower reporting frequency was instead observed compared to other SGLT2 inhibitors (ROR 0.91, 95%CI 0.87–0.96). Considering the higher reporting frequency of ketoacidosis observed with dapagliflozin then DPP-4 inhibitors or insulins, attention should be given to patients treated with this drug.

## 1. Introduction

Dapagliflozin is a selective, highly potent, reversible inhibitor of sodium-glucose cotransporter-2 (SGLT-2), which is responsible for 90% of glucose reabsorption. Through this mechanism, dapagliflozin increases urinary glucose excretion and reduces blood glucose levels [1,2,3]. It is indicated worldwide for the treatment of diabetes mellitus [4]. In particular, in the European Union (EU), it was initially authorized as monotherapy or as add-on combination therapy with other glucose-lowering agents for type 2 diabetes mellitus (T2DM), and lately, in 2019, for overweight patients with type 1 diabetes mellitus (T1DM) not sufficiently controlled with high doses of insulin. The add-on combination therapy is used when the HbA1c is not controlled by monotherapy [5]. Dapagliflozin has represented the first oral treatment approved for T1DM. This decision was based on the results of two Phase III clinical trials of the DEPICT clinical program (Dapagliflozin in Patients with Inadequately Controlled type 1 diabetes mellitus), which demonstrated that dapagliflozin associated with insulin significantly reduced glycated hemoglobin (HbA1c), weight, and total daily insulin dose in T1DM patients not adequately controlled [6]. However, since 25 October 2021, this last therapeutic indication was withdrawn by the manufacturing pharmaceutical industry in agreement with the European Medicine Agency (EMA) and the Health Products Regulatory Authority (HPRA) due to an increased risk of diabetic ketoacidosis observed in this subpopulation [7]. The risk of diabetic ketoacidosis was previously highlighted by clinical trials [8,9,10,11] and then by the Pharmacovigilance Risk Assessment Committee of EMA for the entire drug class of SGLT-2 inhibitors [12]. This risk is the reason that led the US Food and Drug Administration to reject the application of dapagliflozin for T1DM [13]. Diabetic ketoacidosis is a serious and rare complication caused by low insulin levels and high glucagon levels. Early signs and symptoms of diabetic ketoacidosis include polyuria, breathing difficulties, nausea, vomiting and anorexia, excessive thirst, abdominal pain, confusion, unusual asthenia, and sleepiness. More severe symptoms are dehydration, difficulty breathing, confusion, and coma [14,15,16]. Cases of this condition, including life-threatening ones, have occurred in patients taking SGLT2 inhibitors and a number of these cases have been atypical, with patients not having blood sugar levels as high as expected [12]. SGLT2 inhibitors induce ketoacidosis through multiple mechanisms. In the kidney, the inhibition of SGLT2 determines an increase in glycosuria and a reduction of lipolysis with a subsequent increase in ketone reabsorption and blood ketone levels. In the pancreas, as a result of glycosuria and through a direct action on pancreatic α-cells, SGLT2 inhibitors determine an increase in glucagon levels, which induce an increase in hepatic lipolysis and ketogenesis [17,18]. Considering the seriousness of dapagliflozin-induced ketoacidosis and the recent authorization withdrawal for T1DM, we decided to perform a study to describe Individual Case Safety Reports (ICSRs) of ketoacidosis reported in the European database (Eudravigilance, EV) and to assess the reporting frequency of such cases with dapagliflozin.

## 2. Results

During the study period, 2406 out of 9225 ICSRs (26.08%) with dapagliflozin as a suspected drug and reporting at least a ketoacidosis adverse event were retrieved from the EV, of which 200 (8.31%) referred to T1DM, 1191 (49.51%) to T2DM, while in the remaining 42,18% (1015) of ICSRs the indication was not classifiable. The age ranged 18–64 years for all ICSRs (58.69%) and each diabetes group (T1DM 59.00%; T2DM 65.49%; not classifiable 50.64%). Similarly, the gender most reported was feminine for all ICSRs (55.11%) and for each diabetes group (T1DM 67.50%; T2DM 52.31%; not classifiable 55.96%).The 98.79% of all ICSRs was classified as serious. In particular, the 98.50% for T1DM, the 98.57% for T2DM, and the 99.11% for not classifiable ICSRs. The main primary source was the healthcare professional for all ICSRs (94.31%), T1DM (93.50%), T2DM (95.55%), and not classifiable (93.00%). Most ICSRs reported one suspected drug (85.99%) and no concomitant drugs (38.28%). For T2DM, the 28.46% of ICSRs reported more than five concomitant drugs. Characteristics of ICSRs were presented in Table 1. From 2406 ICSRs, we observed a total of 2549 ketoacidosis adverse events (1.06 ADRs per ICSR) since more than one ADR could be reported in each ICSR. Specifically, 213 (8.36%) ketoacidosis adverse events for T1DM, 1275 (50.02%) for T2DM, and 1061 (41.62%) for not classifiable ICSRs. The trend of ketoacidosis adverse events is shown in Figure S1. Most adverse events caused or prolonged hospitalization for all ketoacidosis PTs (50.33%), ketoacidosis PTs in T1DM (46.95%), ketoacidosis PTs in T2DM (52.08%), and ketoacidosis PTs in not classifiable diabetes mellitus (48.92%). The outcome of ketoacidosis adverse events was recovered/resolved for all ketoacidosis PTs (44.72%), ketoacidosis PTs in T1DM (53.99%), and ketoacidosis PTs in T2DM (51.14%), while it was unknown for not classifiable diabetes mellitus (46.28%). Seriousness and outcome criteria for all groups were presented in Table 2.The most reported PT was diabetic ketoacidosis (1412; 55.39%), followed by ketoacidosis (476; 18.67%), euglycaemic diabetic ketoacidosis (296; 11.61%), metabolic acidosis (115; 4.51%), ketonuria (70; 2.75%), ketosis (47; 1.84%), blood ketone body increased (42; 1.65%), urine ketone body present (30; 1.18%), acidosis (23; 0.90%), diabetic ketosis (20; 0.78%), diabetic ketoacidotic hyperglycemic coma (6; 0.24%); blood ketone body present (5; 0.20%), urine ketone body (5; 0.20%), and blood ketone body (2; 0.08%). The PTs specified for the type of diabetes mellitus are reported in Figure S2.

### 2.1. ROR in General Diabetes

**Figure 1:**
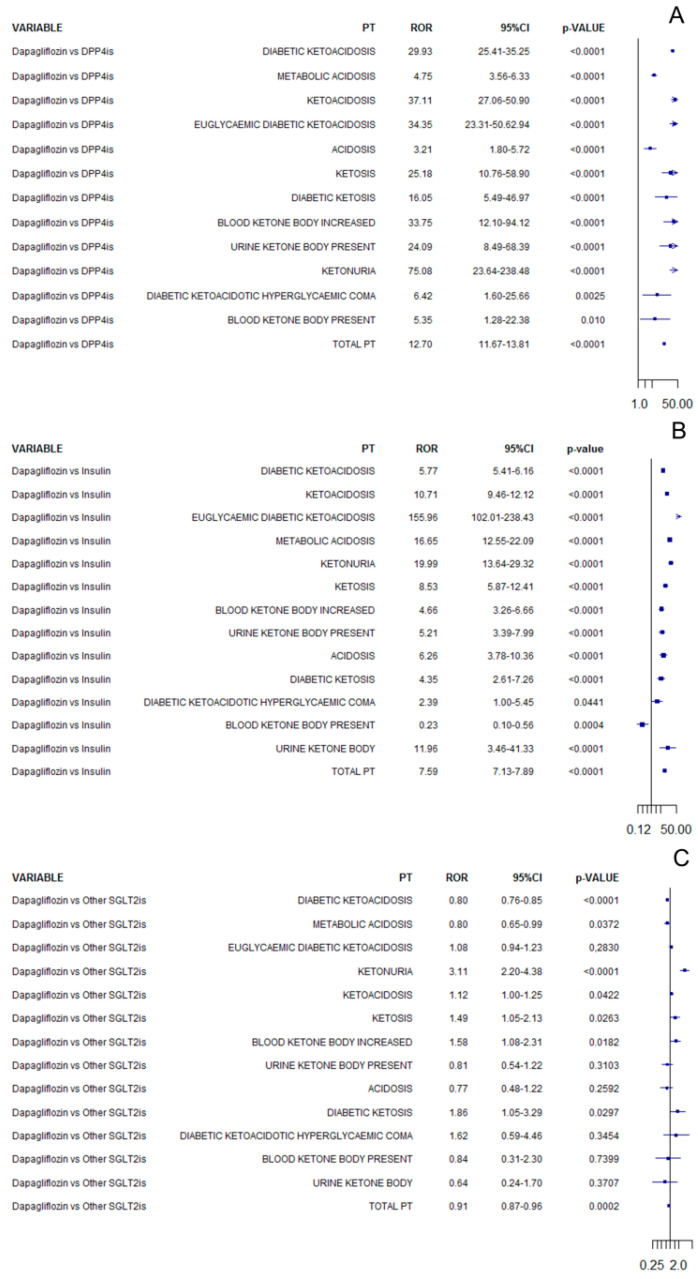
ROR of ketoacidosis adverse events for dapagliflozin compared to DPP-4is (**A**), insulin (**B**), or other SGLT2 inhibitors (**C**) in all cases of diabetes mellitus.

In the analysis considering all ICSRs, dapagliflozin was associated with an increased reporting frequency of ketoacidosis adverse events compared to DPP-4is (ROR 12.07, 95%CI 11.67–13.81; Figure 1A) or insulin (ROR 7.59, 95%CI 7.13–7.89; Figure 1B), while a lower reporting frequency was observed compared to other SGLT2 inhibitors (ROR 0.91, 95%CI 0.87–0.96; Figure 1C). The RORs computed for each specific PT are reported in Figure 1 for all comparisons.

In the direct comparison between SGLT2 inhibitors, no difference was observed when dapagliflozin was compared to canagliflozin (ROR 1.04, 95%CI 0.99–1.10; Figure S3) and ertugliflozin (ROR 1.48, 95%CI 0.95–2.31; Figure S4). On the contrary, a lower reporting frequency was observed when dapagliflozin was compared to empagliflozin (ROR 0.77, 95%CI 0.73–0.82; Figure S5). The RORs related to direct comparisons computed for each specific PT are reported in Figures S3–S5. 

### 2.2. ROR in T1DM

**Figure 2:**
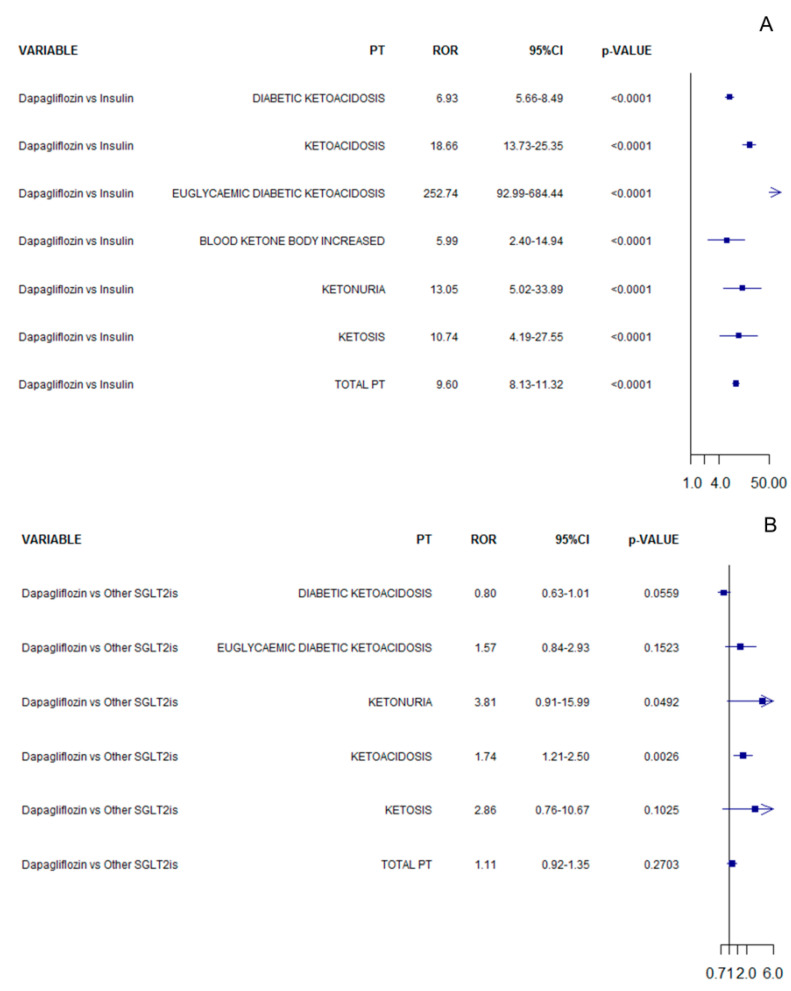
ROR of ketoacidosis adverse events for dapagliflozin compared to insulin (**A**) or other SGLT2 inhibitors (**B**) in T1DM.

In the analysis considering only T1DM ICSRs, dapagliflozin was associated with an increased reporting frequency of ketoacidosis adverse events compared to insulin (ROR 9.60, 95%CI 8.13–11.32; Figure 2A), while no difference was observed compared to other SGLT2 inhibitors (ROR 1.11, 95%CI 0.92–1.35; Figure 2B). The RORs computed for each specific PT are reported in Figure 2 for all comparisons.

In the direct comparison between SGLT2 inhibitors, no difference was observed when dapagliflozin was compared to empagliflozin (ROR 0.91, 95%CI 0.72–1.15; Figure S6), while a lower reporting frequency was observed when dapagliflozin was compared to canagliflozin (ROR 1.26, 95%CI 1.02–1.55; Figure S7).

**Figure 3:**
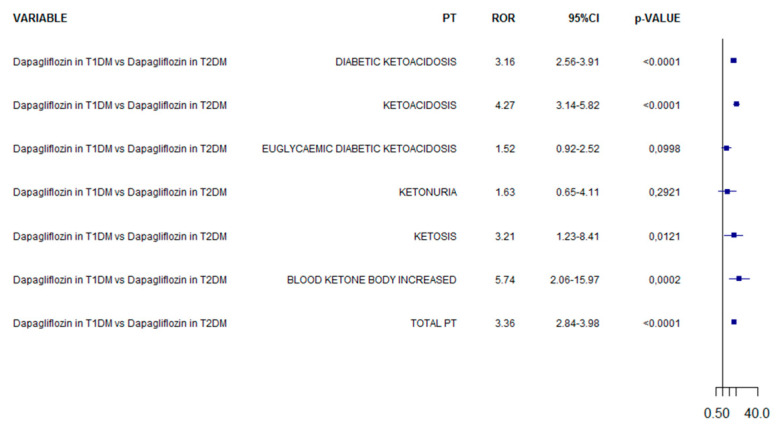
ROR of ketoacidosis adverse events for dapagliflozin in T1DM compared to dapagliflozin in T2DM.

In the analysis comparing dapagliflozin in T1DM vs. dapagliflozin in T2DM, the reporting frequency was increased of 3.36-fold in T1DM (95%CI 2.84–3.98; Figure 3). The RORs computed for each specific PT are reported in Figure 3.

## 3. Discussion

The present study evaluated the effect of dapagliflozin on ketoacidosis adverse events by using data from the EV database. Our main result reflects a higher reporting frequency of ketoacidosis adverse events with dapagliflozin, supporting in part the risk observed in other observational studies [19,20,21]. However, data reported in the EV database concerns suspected ADRs that have been observed following the use of a drug, but which are not necessarily related to or caused by the drug. 

In our general descriptive analysis, we found that about 26% of all ICSRs of dapagliflozin reported at least one ketoacidosis adverse event and that most of them occurred in T2DM patients (49.51%). This result is in line with the most recent marketing authorization of dapagliflozin for T2DM that happened about 7 years earlier than that of T1DM. According to our results, ketoacidosis adverse events occurred most frequently in female patients, enough to be considered among risk factors for diabetic ketoacidosis [22]. Lastly, it should be noted that women are more susceptible to experience ADRs due to hormones and changes in pharmacokinetics, independently from the drug class [23,24,25]. Moreover, we observed most ICSRs in patients aged 18–64 years in line with marketing authorization details that stated the use only in adult patients [26]. However, results in the literature are conflicting and an observational study found that age and sex did not modify the association of dapagliflozin and ketoacidosis [19].

The 94.31% of ICSRs were reported by HCPs in accordance with other studies [27,28,29,30,31]. In most ICSRs was reported one suspected drug and no concomitant drugs. This may imply a low presence of contributing factors to the occurrence of adverse events, but the incompleteness of information should be considered as well. 

Most ICSRs were classified as serious due to hospitalization and because the most reported events (such as diabetic ketoacidosis, ketoacidosis, and euglycaemic diabetic ketoacidosis) are included in the Important Medical Event (IME) List 24.1 of EMA. Moreover, ketoacidosis adverse events result in a complete resolution in most ICSRs despite a little more of 30% had an unknown outcome that could affect the proportion of cases with a complete resolution that, therefore, cannot be perfectly estimated. Accordingly, in the literature, patients are used to restart the treatment with SGLT2 inhibitors following the resolution of the event [32]. However, we could not establish with our data if the treatment was restarted again due to our study limitations. Knowing the outcome is also important to better understand the impact of the higher reporting frequency of ketoacidosis events on the patients’ quality of life.

In the analysis related to all diabetes ICSRs, dapagliflozin was associated with a 12.7- and 7.6-fold increased reporting frequency of ketoacidosis adverse events compared to DPP-4is and insulin, respectively. Considering literature data, previous studies on spontaneous reporting system also found a high reporting frequency of ketoacidosis associated with SGLT2 inhibitors [33,34]. However, when the risk of ketoacidosis was evaluated in terms of hazard, observational studies found lower estimates. Specifically, a population-based cohort study comparing patients with T2DM who are new users of SGLT-2 inhibitors with those receiving DPP-4is showed a Hazard Ratio of 2.85 (95%CI 1.99–4.08) [19]. Moreover, a nationwide register-based cohort study from two countries found that the use of SGLT2 inhibitors compared with Glucagon Like Peptide-1 receptor agonists was associated with a 2.1-fold increased risk of diabetic ketoacidosis (95%CI 1.01–4.52) in T2DM patients [21]. Furthermore, a meta-analysis of randomized trials and cohort studies found a 2.5-(95%CI 1.16–5.21) and 1.7-fold (95%CI 1.07–2.83) higher risk of diabetic ketoacidosis with SGLT2 inhibitors compared with reference groups, respectively [35]. These differences in the estimates may be due to the different study design and analysis.

Dapagliflozin used for T1DM was associated with a 3.4-fold increased reporting frequency of ketoacidosis adverse events compared with dapagliflozin used for T2DM. In the analysis considering only T1DM ICSRs, we found a reporting frequency of ketoacidosis adverse events for dapagliflozin compared to insulins that was higher than that observed in all diabetes cases. Indeed, it was increased 9.6-fold compared to 7.6-fold observed in all cases. The increased frequency observed in T1DM can be explained by the renal and pancreatic effects of SGLT2-inhibitors that are emphasized in patients in add-on combination therapy with insulin, since to minimize the risk of hypoglycemia it is necessary to decrease the insulin dose. This furtherly alters the glucagon/insulin ratio contributing to an additional increase in circulating ketone body levels [36]. This mechanism is probably exploit for their use in heart failure [37], since ketones can be cardiac substrates that improve the metabolic efficiency of the heart [4]. Furthermore, it should be considered that diabetic ketoacidosis is caused by low levels of insulin and that the administration of intravenous insulin is frequently used to resolve this adverse event [38]. Indeed, to prevent the potentially dangerous complications associated with diabetes ketoacidosis, patients in treatment with SGLT2 inhibitors who develop the event should discontinue the medication, undergo ketone evaluation, and start insulin therapy, if blood ketones are detected [39]. The risk in T1DM was assessed in previous clinical trials. The Dapagliflozin Evaluation in Patients with Inadequately Controlled T1DM (DEPICT-2) trial found that diabetic ketoacidosis was more reported in the dapagliflozin groups compared to placebo with the majority of events that were mild or moderate in severity and that resolved after treatment [11]. Similarly, the pooled analysis of DEPICT-1 and DEPICT-2 studies found an increased onset of diabetic ketoacidosis with dapagliflozin compared with placebo over 52 weeks [40]. However, a recent meta-analysis of randomized controlled clinical trials seem to find that the short-term use (24 weeks) of dapagliflozin associated with insulin for T1DM was not associated with an increased risk of diabetic ketoacidosis, but as highlighted by Authors additional high-quality studies are needed to determine its long-term safety [41]. However, the risk of ketoacidosis associated SGLT2 inhibitors has led over the years to recommendations for the management of this event in patients with T1DM [42]. Finally, a lower reporting frequency was observed when dapagliflozin was compared to other SGLT2 inhibitors in all diabetes cases, while no difference was showed in T1DM cases. This result may highlight that these drugs share the same mechanism of action suggesting a drug class effect [19]. Indeed, cases of ketoacidosis have been also reported for the other SGLT2 inhibitors [43,44,45]. Moreover, this may influence the choice of a drug from this therapeutic class but further studies are needed to confirm this hypothesis.

### Strengths and Limitations

Our study has some strengths and limitations. Among the strengths, we should consider that the spontaneous reporting system is a useful and inexpensive tool for the collection and analysis of drugs safety data and the better characterization of the drug safety profiles. Indeed, through data from the spontaneous reporting system, we can detect ADRs not detectable during the pre-marketing phase, including rare and serious ones. Therefore, through the EV database, we have analyzed a large amount of ICSRs related to dapagliflozin and the entire European area. Among limitations, there is the under-reporting and the poor quality of information listed in each ICSR. In particular, under-reporting is a major limitation of spontaneous reporting systems that reduces sensitivity because it underestimates the frequency and the impact of a given ADRs. Moreover, the incompleteness of information reported in the ICSRs could have affected our results by influencing the case selection based on the types of diabetes mellitus. Another limitation of our study is that we cannot establish causality with our data. Finally, an important specific limitation for the SGLT2 inhibitors as well as for other glucose-lowering therapies with cardio-metabolic benefits is related to the barriers of prescribing such as costs and unfamiliarity with their use that could have limited study results [46,47,48]. 

## 4. Materials and Methods

### 4.1. Study Design

The DAPAgliflozin and KETOacidosis (DAPA-KETO) study is a systematic analysis of the European pharmacovigilance database (EudraVigilance).

### 4.2. Data Source

Data on ICSRs with dapagliflozin as a suspected drug were retrieved from the website of suspected adverse drug reactions (ADRs) of EV (www.adrreports.eu, accessed on 21 September 2021). The EV is supervised by the EMA and it is a system used for the management and analyses of ICSRs related to both medicines or vaccines, which are authorized or are being evaluated in clinical trials within the European Economic Area (EEA). The EV contains all ICSRs reported by a healthcare professional (HCP) or a non-HCP to an EU national competent authority or a marketing authorization holder. These data are publicly available for transparency through the EMA website (www.adrreports.eu, accessed on 21 September 2021). 

### 4.3. ICSRs Selection with Line Listing

By using the line listing function, ICSRs reporting dapagliflozin as a suspected drug were retrieved from the date of marketing authorization granted by the EMA to 11 September 2021. According to the Assessment Report of SGLT2 inhibitors published by the EMA [49], we identified a case of ketoacidosis by using the following preferred terms (PTs) of the Medical Dictionary for Regulatory Activities (MedDRA): acidosis, blood ketone body present, blood ketone body increased, blood ketone body, diabetic ketoacidosis, diabetic ketoacidotic hyperglycemic coma, diabetic ketosis, euglycaemic diabetic ketoacidosis, ketoacidosis, ketonuria, ketosis, metabolic acidosis, urine ketone body present, and urine ketone body. MedDRA terminology is described elsewhere [29,50].

### 4.4. Descriptive Analyses

Information on patient characteristics (age and gender), the seriousness of the adverse event, primary source qualification, primary source country for regulatory purposes, number of suspected drugs other than dapagliflozin, and number of concomitant drugs were provided for all ICSRs. ICSRs were also classified and summarized based on the therapeutic indication in those related to T1DM or T2DM. If the dapagliflozin therapeutic indication was not specific, the ICSRs were defined as not classifiable for the type of diabetes and described separately. 

In accordance with the International Council on Harmonization E2D guidelines, we classified the seriousness of a ketoacidosis adverse event as life-threatening, results in death, caused/prolonged hospitalization, disabling, determines a congenital anomaly/birth defect, or other medically important condition. If more than one criterion of seriousness was reported for an adverse event, we chose for classification the most serious. The outcome of a ketoacidosis adverse event was classified as “Recovered/Resolved”, “Recovering/Resolving”, “Recovered/Resolved With Sequelae”, “Not Recovered/Not Resolved”, “Fatal”, and “Unknown. The outcome with the lower level of resolution was chosen for classification whether an adverse event of ketoacidosis reported two or more different outcomes. Ketoacidosis adverse events of dapagliflozin were summarized for each type of diabetes. 

### 4.5. Descriptive Analyses

The Reporting Odds Ratio (ROR) and its’ 95% confidence interval (95%CI) was computed to assess the reporting frequency of ketoacidosis adverse events for dapagliflozin compared to dipeptidyl peptidase-4 inhibitors (DPP-4is) or insulins. We chose DPP4is as the comparator group because they are a second-line treatment similarly used for diabetes and have no known association with diabetic ketoacidosis [51]. While we used insulins because they are widely used for T1DM. 

Moreover, a sub-analysis was performed to compare dapagliflozin with all other SGLT2 inhibitors, and with each specific SGLT2 inhibitor. Single drugs considered in reference groups are listed in Table S1. For drugs used in both diabetes forms, analyses were performed on all ICSRs retrieved from the EV website and separately for those classified as T1DM. Finally, the ROR was computed to evaluate the reporting frequency of ketoacidosis adverse events with dapagliflozin used in T1DM compared to its use in T2DM. RORs were carried out for PTs reported at least in a number of cases ≥3, and also for the sum of all PTs of ketoacidosis (Total PT). Data management and analysis were performed with Microsoft Excel 2019 and R (version 3.2.2, R Development Core Team). Forest plots were performed using R (version 3.2.2, R Development Core Team).

## 5. Conclusions

We carried out descriptive and statistical analyses from 2406 ICSRs (covering 2549 ketoacidosis ADRs) related to dapagliflozin. Ketoacidosis ADRs were serious in more than 98% of cases and the outcome was resolved in 44% of cases. The most reported ketoacidosis adverse events were diabetic ketoacidosis, ketoacidosis, and euglycaemic diabetic ketoacidosis. Applying the ROR, we found that dapagliflozin was associated with a higher reporting frequency of ketoacidosis when compared to DPP4is or insulin in all analyses. No difference was instead observed in the comparison with all other SGLT2 inhibitors for all analyses. The higher risk of ketoacidosis with dapagliflozin has led to the marketing authorization withdrawal for T1DM. However, since dapagliflozin remains authorized for T2DM and that the risk of ketoacidosis can be associated with all SGLT2 inhibitors, attention should be given to patients treated with these drugs. Indeed, patients in therapy with SGLT2 inhibitors must contact their medical doctors if they have symptoms such as rapid weight loss, nausea or vomiting, stomach pain, excessive thirst, fast and deep breathing, confusion, unusual sleepiness, and others. Moreover, the occurrence of this adverse event in the setting of relative euglycemia could be critical for recognizing this life-threatening diabetes complication. Ketoacidosis is being increasingly reported with SGLT2 inhibitors since these drugs are prescribed in the primary care setting. In conclusion, given the potential seriousness of dapagliflozin-induced ketoacidosis and considering the intrinsic limitations of our study, we believe that further high-quality clinical studies should be conducted on this topic, especially in T2DM patients, to better estimate the impact of SGLT2 inhibitors on diabetic ketoacidosis.

## Figures and Tables

**Table 1:** Distribution for age, gender, seriousness, primary source, primary source country, number of suspected drugs, and number of concomitant drugs of Individual Case Safety Reports (ICSRs) reporting at least one event classifiable as ketoacidosis and having dapagliflozin as suspected drug among those reported in Eudravigilance from the date of marketing authorization to 11 September 2021.

Variable	Level	All ICSRs (*n* = 2406)	ICSRs in T1DM (*n* = 200)	ICSRs in T2DM (*n* = 1191)
Age	<18 years (%)	8 (0.33)	8 (4.00)	0 (0)
	18–64 years (%)	1412 (58.69)	118 (59.00)	780 (65.49)
	>65 years (%)	443 (18.41)	17 (8.50)	233 (19.56)
	Missing (%)	543 (22.57)	57 (28.50)	178 (14.95)
Gender	F (%)	1326 (55.11)	135 (67.50)	623 (52.31)
	M (%)	1001 (41.60)	52 (26.00)	543 (45.59)
	Missing (%)	79 (3.28)	13 (6.50)	25 (2.10)
Seriousness of ICSR	Serious (%)	2377 (98.79)	197 (98.50)	1174 (98.57)
	Not serious (%)	29 (1.21)	3 (1.50)	17 (1.43)
Primary Source	Healthcare Professional (%)	2269 (94.31)	187 (93.50)	1138 (95.55)
	Non-Healthcare Professional (%)	137 (5.69)	13 (6.50)	53 (4.45)
Primary Source Country for Regulatory Purposes	European Economic Area (%)	1010 (41.98)	71 (35.50)	547 (45.93)
	Non-European Economic Area (%)	1396 (58.02)	129 (64.50)	644 (54.07)
Number of Suspected drug(s)	1 (%)	2069 (85,99)	163 (81.50)	1015 (85.22)
	2 (%)	228 (9.48)	29 (14.50)	113 (9.49)
	3 (%)	72 (2.99)	6 (3.00)	39 (3.27)
	4 (%)	12 (0.50)	1 (0.50)	9 (0.76)
	≥5 (%)	25 (1.04)	1 (0.50)	15 (1.26)
Number of Concomitant drug(s)	0 (%)	921 (38.28)	74 (37.00)	330 (27.71)
	1 (%)	301 (12.51)	47 (23.50)	138 (11.59)
	2 (%)	320 (13.30)	28 (14.00)	181 (15.20)
	3 (%)	211 (8.77)	20 (10.00)	116 (9.74)
	4 (%)	143 (5.94)	11 (5.50)	87 (7.30)
	≥5 (%)	510 (21.20)	20 (10.00)	339 (28.46)

**Table 2:** Seriousness and outcome of ketoacidosis adverse events distributed by type of diabetes and having dapagliflozin as suspected drug among those reported in Eudravigilance from the date of marketing authorization to 11 September 2021.

Variable	Level	All ketoacidosis PT (*n* = 2549)	Ketoacidosis PT in T1DM (*n* = 213)	Ketoacidosis PT in T2DM (*n* = 1275)
Seriousness	Caused/Prolonged Hospitalization (%)	1283 (50.33)	100 (46.95)	664 (52.08)
	Other Medically Important Condition (%)	665 (26.09)	53 (24.88)	256 (20.08)
	Life Threatening (%)	510 (20.01)	50 (23.47)	304 (23.84)
	Results in Death (%)	45 (1.77)	6 (2.82)	23 (1.80)
	Not Serious (%)	35 (1.37)	4 (1.88)	22 (1.73)
	Disabling (%)	11 (0.43)	0 (0.00)	6 (0.47)
Outcome	Recovered/Resolved (%)	1140 (44.72)	115 (53.99)	652 (51.14)
	Unknown (%)	897 (35.19)	64 (30.05)	342 (26.82)
	Recovering/Resolving (%)	374 (14.67)	18 (8.45)	204 (16.00)
	Not Recovered/Not Resolved (%)	93 (3.65)	13 (6.10)	51 (4.00)
	Fatal (%)	30 (1.18)	2 (0.94)	17 (1.33)
	Recovered/Resolved With Sequelae (%)	15 (0.59)	1 (0.47)	9 (0.71)

## Data Availability

European Pharmacovigilance data are available at www.adrreports.eu (accessed on 21 September 2021).

## Supplementary Materials

The following supporting information can be downloaded at: https://www.mdpi.com/article/10.3390/ph15030286/s1, Table S1: Drugs included in reference groups; Figure S1: Trend of ketoacidosis adverse events for each type of diabetes mellitus; Figure S2: ketoacidosis adverse events for type of diabetes mellitus; Figure S3: ROR of ICSRs with ketoacidosis adverse events for dapagliflozin compared to canagliflozin in all diabetes mellitus cases; Figure S4: ROR of ICSRs with ketoacidosis adverse events for dapagliflozin compared to ertugliflozin in all diabetes mellitus cases; Figure S5: ROR of ICSRs with ketoacidosis adverse events for dapagliflozin compared to empagliflozin in all diabetes mellitus cases; Figure S6: ROR of ICSRs with ketoacidosis adverse events for dapagliflozin compared to empagliflozin in T1DM; Figure S7: ROR of ICSRs with ketoacidosis adverse events for dapagliflozin compared to canagliflozin in T1DM.
