# Supplementary material for: The Reporting Frequency of Ketoacidosis Events with Dapagliflozin from the European Spontaneous Reporting System: The DAPA-KETO Study
Source: Pharmaceuticals (Basel). 2022 Feb 25;15(3):286. doi: 10.3390/ph15030286 (PMC8952809; doi:10.3390/ph15030286)
Supplement: Supplementary file 1 [file pharmaceuticals-15-00286-s001.zip › pharmaceuticals-1600543-supplementary.pdf]

SUPPLEMENTARY MATERIAL

RESULTS

Table S1. Drugs included in reference groups.

Reference group	Drugs
Dipeptidyl peptidase-4 inhibitors (DPP-4is)	Alogliptin, linagliptin, saxagliptin, sitagliptin, and vildagliptin
Insulins	Insulin aspart, bovine insulin, insulin degludec, insulin detemir, insulin glargine, insulin glulisine, human insulin, insulin lispro, insulin glargine/lixisenatide, porcine insulin, insulin zinc, and insulin aspart/ insulin degludec
Other SGLT2-inhibitors	Canagliflozin, empagliflozin, and ertugliflozin

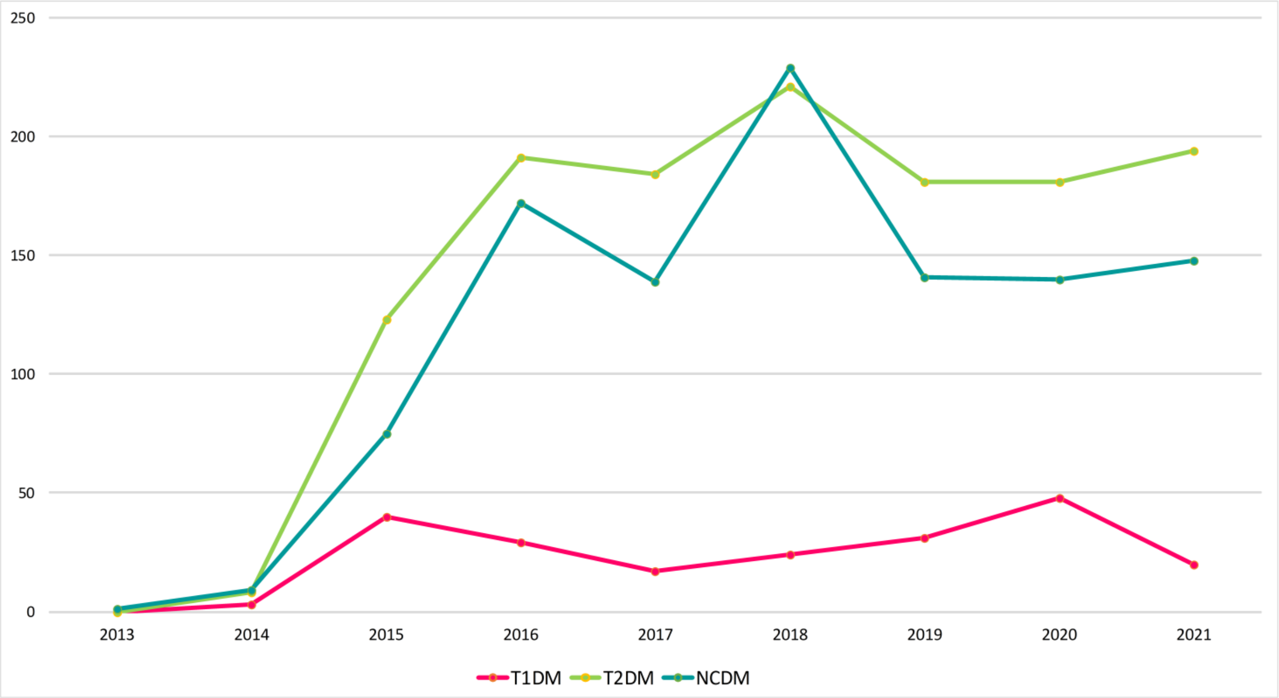

Figure S1. Trend of ketoacidosis adverse events with dapagliflozin for each type of diabetes mellitus.

*No case of ketoacidosis was reported in 2012

T1DM: type 1 diabetes mellitus, T2DM: type 2 diabetes mellitus, NCDM: not classifiable diabetes mellitus

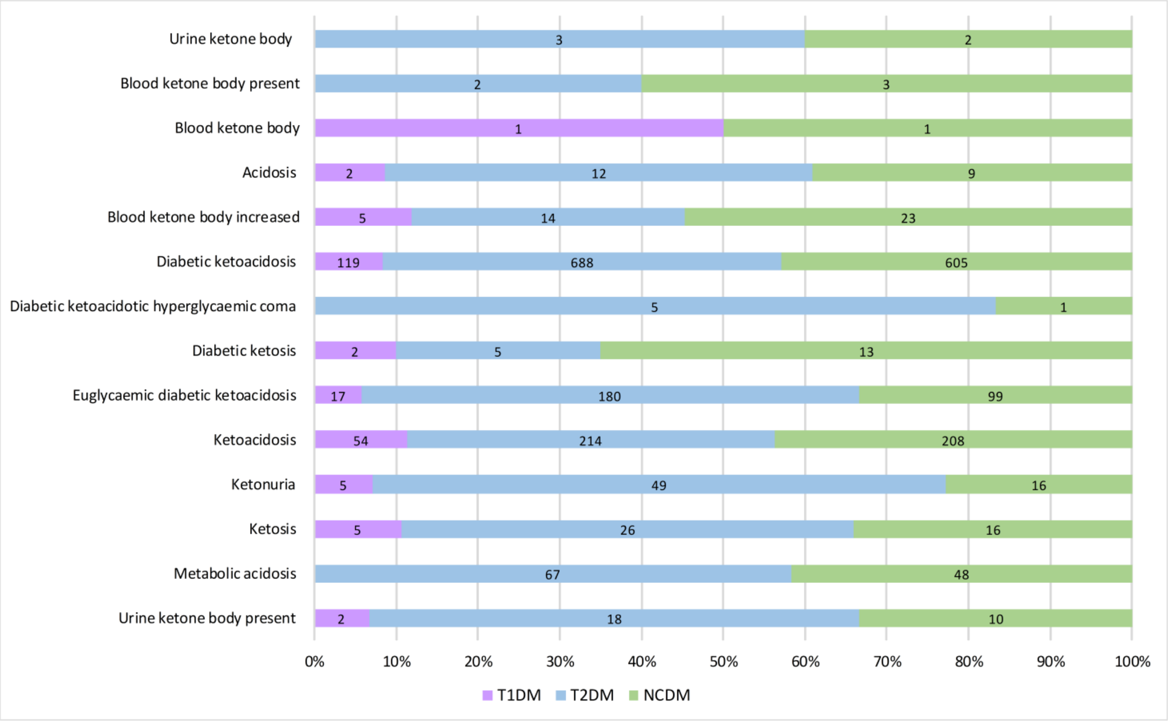

Figure S2. ketoacidosis adverse events for type of diabetes mellitus.
T1DM: type 1 diabetes mellitus, T2DM: type 2 diabetes mellitus, NCDM: not classifiable diabetes mellitus

VARIABLE	PT	ROR	95%CI	p-VALUE
Dapagliflozin vs canagliflozin	DIABETIC KETOACIDOSIS	0.78	0.73-0.84	<0.0001
Dapagliflozin vs canagliflozin	METABOLIC ACIDOSIS	0.76	0.60-0.95	0.0139
Dapagliflozin vs canagliflozin	EUGLYCAEMIC DIABETIC KETOACIDOSIS	3.01	2.47-3.65	<0.0001
Dapagliflozin vs canagliflozin	KETONURIA	4.21	2.68-6.61	<0.0001
Dapagliflozin vs canagliflozin	KETOACIDOSIS	1.56	1.38-1.78	<0.0001
Dapagliflozin vs canagliflozin	KETOSIS	3.06	1.87-5.01	<0.0001
Dapagliflozin vs canagliflozin	BLOOD KETONE BODY INCREASED	3.28	1.93-5.59	<0.0001
Dapagliflozin vs canagliflozin	URINE KETONE BODY PRESENT	0.69	0.45-1.06	0.0866
Dapagliflozin vs canagliflozin	ACIDOSIS	1.09	0.64-1.85	0.7563
Dapagliflozin vs canagliflozin	DIABETIC KETOSIS	3.90	1.72-8.87	0.0004
Dapagliflozin vs canagliflozin	DIABETIC KETOACIDOTIC HYPERGLYCAEMIC COMA	1.87	0.57-6.14	0.2921
Dapagliflozin vs canagliflozin	URINE KETONE BODY	1.56	0.45-5.39	0.4778
Dapagliflozin vs canagliflozin	BLOOD KETONE BODY	0.62	0.12-3.22	0.5698
Dapagliflozin vs canagliflozin	TOTAL PT	1.04	0.99-1.10	0.1121

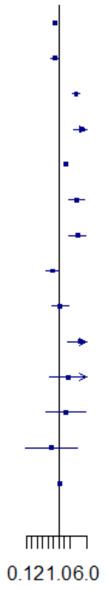

Figure S3. ROR of ICSRs with ketoacidosis adverse events for dapagliflozin compared to canagliflozin in all diabetes mellitus cases.

VARIABLE	PT	ROR	95%CI	p-VALUE
Dapagliflozin vs ertugliflozin	DIABETIC KETOACIDOSIS	1.20	0.70-2.05	0.5154
Dapagliflozin vs ertugliflozin	EUGLYCAEMIC DIABETIC KETOACIDOSIS	0.69	0.28-1.69	0.4143
Dapagliflozin vs ertugliflozin	TOTAL PT	1.48	0.95-2.31	0.0847

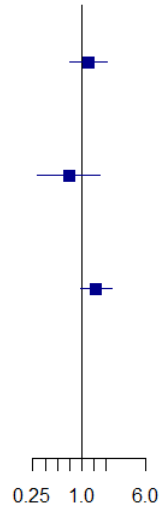

Figure S4. ROR of ICSRs with ketoacidosis adverse events for dapagliflozin compared to ertugliflozin in all diabetes mellitus cases.

VARIABLE	PT	ROR	95%CI	p-VALUE
Dapagliflozin vs empagliflozin	DIABETIC KETOACIDOSIS	0.83	0.77-0.89	<0.0001
Dapagliflozin vs empagliflozin	METABOLIC ACIDOSIS	0.87	0.68-1.11	0.2734
Dapagliflozin vs empagliflozin	EUGLYCAEMIC DIABETIC KETOACIDOSIS	0.57	0.50-0.66	<0.0001
Dapagliflozin vs empagliflozin	KETONURIA	2.28	1.52-3.43	<0.0001
Dapagliflozin vs empagliflozin	KETOACIDOSIS	0.80	0.71-0.90	0.0002
Dapagliflozin vs empagliflozin	KETOSIS	0.88	0.60-1.28	0.5012
Dapagliflozin vs empagliflozin	BLOOD KETONE BODY INCREASED	0.92	0.61-1.38	0.6882
Dapagliflozin vs empagliflozin	URINE KETONE BODY PRESENT	1.07	0.65-1.76	0.7953
Dapagliflozin vs empagliflozin	ACIDOSIS	0.55	0.33-0.90	0.0153
Dapagliflozin vs empagliflozin	DIABETIC KETOSIS	1.09	0.59-2.00	0.7937
Dapagliflozin vs empagliflozin	DIABETIC KETOACIDOTIC HYPERGLYCAEMIC COMA	1.37	0.42-4.48	0.6040
Dapagliflozin vs empagliflozin	BLOOD KETONE BODY PRESENT	0.41	0.15-1.13	0.0743
Dapagliflozin vs empagliflozin	URINE KETONE BODY	0.36	0.13-0.97	0.0351
Dapagliflozin vs empagliflozin	BLOOD KETONE BODY	0.13	0.03-0.58	0.0016
Dapagliflozin vs empagliflozin	TOTAL PT	0.77	0.73-0.82	<0.0001

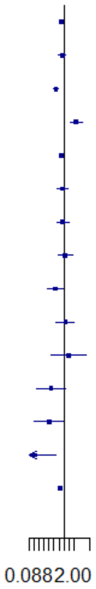

Figure S5. ROR of ICSRs with ketoacidosis adverse events for dapagliflozin compared to empagliflozin in all diabetes mellitus cases.

VARIABLE	PT	ROR	95%CI	p-VALUE
Dapagliflozin vs Empagliflozin in T1DM	DIABETIC KETOACIDOSIS	0.88	0.66-1.17	0.3845
Dapagliflozin vs Empagliflozin in T1DM	EUGLYCAEMIC DIABETIC KETOACIDOSIS	0.67	0.35-1.28	0.2183
Dapagliflozin vs Empagliflozin in T1DM	KETOACIDOSIS	1.05	0.69-1.59	0.8162
Dapagliflozin vs Empagliflozin in T1DM	KETOSIS	1.39	0.33-5.85	0.6501
Dapagliflozin vs Empagliflozin in T1DM	BLOOD KETONE BODY INCREASED	1.39	0.33-5.85	0.6501
Dapagliflozin vs Empagliflozin in T1DM	TOTAL PT	0.91	0.72-1.15	0.4160

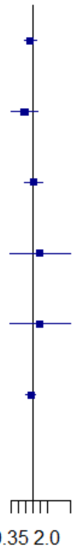

Figure S6. ROR of ICSRs with ketoacidosis adverse events for dapagliflozin compared to empagliflozin in T1DM.

VARIABLE	PT	ROR	95%CI	p-VALUE
Dapagliflozin vs Canagliflozin in T1DM	DIABETIC KETOACIDOSIS	0.76	0.59-0.97	0.0265
Dapagliflozin vs Canagliflozin in T1DM	EUGLYCAEMIC DIABETIC KETOACIDOSIS	8.34	2.44-28.57	<0.0001
Dapagliflozin vs Canagliflozin in T1DM	KETOACIDOSIS	2.72	1.72-4.30	<0.0001
Dapagliflozin vs Canagliflozin in T1DM	TOTAL PT	1.26	1.02-1.55	0.0354

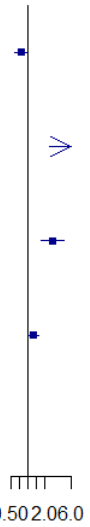

Figure S7. ROR of ICSRs with ketoacidosis adverse events for dapagliflozin compared to canagliflozin in T1DM.
